# Use of serum C-reactive protein as an early marker of inflammatory activity in canine type II immune-mediated polyarthritis: case report

**DOI:** 10.1186/1751-0147-48-9

**Published:** 2006-06-21

**Authors:** Mads Kjelgaard-Hansen, Asger Lundorff Jensen, Geoffrey A Houser, Lisbeth Rem Jessen, Annemarie T Kristensen

**Affiliations:** 1 Central Laboratory, Department of Small Animal Clinical Sciences, The Royal Veterinary and Agricultural University. 3 Gronnegaardsvej, DK-1870 Frederiksberg, Denmark; 2 Small Animal Veterinary Teaching Hospital, Department of Small Animal Clinical Sciences, The Royal Veterinary and Agricultural University. 16 Dyrlaegevej, DK-1870 Frederiksberg, Denmark

## Abstract

**Background:**

Monitoring systemic inflammatory activity during steroid therapy of canine immune-mediated polyarthritis (IMPA) is difficult and mainly relies on clinical signs.

**Case presentation:**

Canine serum C-reactive protein (CRP) was measured serially and blinded during a 27-week follow-up period of a case of *Anaplasma phagocytophilia *induced type II immune-mediated polyarthritis.

**Conclusion:**

WBC was, as expected, observed not to reflect the inflammatory activity during steroid treatment in a clinical useful manner, whereas, CRP is suggested a valuable unbiased marker of inflammatory activity during steroid treatment in this case.

## Background

Monitoring systemic inflammatory activity during steroid therapy of canine immune-mediated diseases may be difficult. The clinician mainly has to rely on clinical signs [1] since objective fast-reacting inflammatory markers (white blood cell count [WBC] and absolute numbers of granulocytes) usually are so affected by the steroid treatment *per se *[2] that they are inadequate for reliable monitoring. Thus, a fast-reacting objective inflammatory marker not biased by steroids could potentially be of clinical value.

One such marker could be canine C-reactive protein (CRP). Studies on canine CRP reported clinical applicability for monitoring variation in inflammatory activity during various stages of disease [3,4], assessing therapy efficiency[4,5] and was reported to be unbiased by corticosteroids[6,7]. Furthermore, validated assays for measuring canine CRP are commercially available [8-10]. This report describes a case of canine type II IMPA that was monitored blinded in the follow-up period using serial measurements of canine serum CRP concentration.

## Case report

### Diagnosis

A 9-year old, female English Springer Spaniel was referred to the Small Animal Veterinary Teaching Hospital, Department of Small Animal Clinical Sciences, The Royal Veterinary and Agricultural University, Denmark with a history of weight-loss, lethargy, intermittent lameness, generalised lymphadenopathy and recurrent febrile episodes during the preceding 8 weeks despite antibiotic and anti-inflammatory steroid treatment. Clinical investigation revealed depression, pyrexia (39.9°C), lameness, reluctance to stand and joint pain in multiple joints. Diagnostic procedures included complete blood count (CBC), blood smear analysis, serum biochemistry, urinalysis, cytological evaluation of lymph nodes and synovial fluid and radiographs of joints. The CBC, blood smear and serum biochemistry revealed a regenerative anaemia characterised by increased reticulocyte count, spherocytosis and erythrocyte autoagglutination. Cytology revealed reactive lymphadenopathy in lymph notes and neutrophilic inflammation in all joints sampled, with *Anaplasma phagocytophilum*-like inclusions in occasional neutrophils. Radiographs revealed no sign of erosive joint-processes with only slight soft tissue changes. A diagnosis of type II Immune-mediated polyarthritis (IMPA) and immune-mediated haemolytic anaemia (IMHA) was established and antibiotic therapy (doxycycline 10 mg/kg sid [Ronaxan; Merial]) was initiated. To further confirm *A. phagocytophilum *infection and rule out other potential suspect causes of IMHA and type II IMPA, thoracic radiographs, abdominal ultrasound, PCR tests for canine distemper virus, *Ehrlichia spp*., serum antibody titer-tests for *Borrelia spp., Bartonella spp*. and *Babesia spp*. and anti-nuclear antibody test were performed. All were unremarkable. A serum antibody titer for *Ehrlichia equi *(*Anaplasma phagocytophilum *[11]) was, however, positive (IgG titer 1:640 [cut-off; 1:32]). Based on the clinical and paraclinical examinations, the dog was considered to suffer from IMHA and a type II IMPA secondary to an *A. phagocytophilum *infection.

Immunosuppressive therapy (prednisolone 1.0 mg/kg bid [Prednisolonacetat; Nycomed]) was initiated and antibiotic therapy (doxycycline 10 mg/kg sid) was continued.

### Follow-up

**Figure 1:**
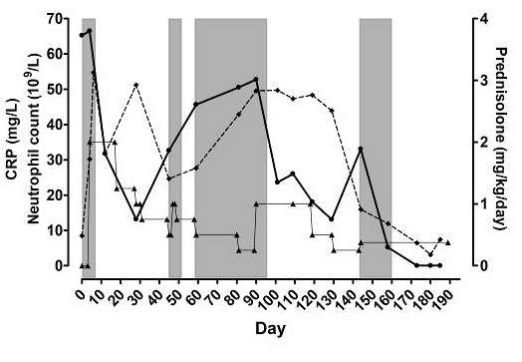
Neutrophil count [◆], serum C-reactive protein concentrations (CRP [●]) and daily dosages of prednisolone [▲] in a dog with type II immune-mediated polyarthritis plotted against days after referral of patient. Shaded areas are periods with clinical symptoms of polyarthritis.

In the follow-up period, the dog was monitored by means of clinical examinations and CBC on a weekly to bi-weekly schedule. C-reactive protein were measured by means of a validated human CRP immunoturbidimetric assay [8,12] in parallel with CBC. The CRP values were not disclosed to the clinicians (blinded). The corticosteroid dosage was attempted titrated to an acceptable clinical outcome regarding symptoms of the IMPA and adverse effects of therapy (Fig. 1). The dog had several periods with relapse of clinical symptoms of polyarthritis (Fig. 1) mainly in relation to tapering of the corticosteroid therapy. Azathioprine (2.0 mg/kg/day [Imurel; Glaxo Wellcome]) was included in the therapy regimen from day 105 in combination with prednisolone to possibly lower the necessary dose of prednisolone (clinical signs of steroid associated adverse effects [polyuria, polydipsia, panting and Cushingoid appearence] were observed at the dosage needed for sufficient IMPA suppression). For 38 days no clinical signs of IMPA were observed on a combination of prednisolone (0.125-0.5 mg/kg bid) and azathioprine (2.0 mg/kg sid), with acceptable degrees of adverse effects at the lower doses of prednisolone. Symptoms of IMPA returned at day 143. Bone marrow suppression with panleukopenia (WBC<6.5·10^9^/L) (Fig. 1) and thrombocytopenia (Thrombocyte count<200·10^9^/L) was observed at day 170, where the azathioprine dosage was halved and later discontinued at day 180. The dog was euthanized at day 189 as the owner declined further therapy. Necropsy was not performed, due to owner's reluctance. The WBC and neutrophil count were abnormally high from initiation of therapy until day 144 and 158, respectively and decreased to leukopenic and neutropenic (<3.2·10^9^/L) levels at day 170. The observed fluctuations of the WBC paralleled the fluctuations of the neutrophil count very closely and the data are therefore not shown. Both parameters did not reflect the clinical signs of disease activity in a manner useful for treatment decisions (see neutrophil count in fig. 1).

### CRP variations during therapy

High CRP concentrations (local reference interval 0–18 mg/L) were observed at day 0–4 (65–66 mg/L) declining to within reference interval during initial high-dose prednisolone treatment (0.5–1.0 mg/kg bid [day 5–30]) (Fig. 1). From day 30 to day 90 (with prednisolone dosage consistently below 0.5 mg/kg bid) CRP gradually increased. The CRP was already increased at the first relapses of clinical signs of polyarthritis (day 47–49 and day 61–95) and CRP was persistently high during the periods of clinical signs. An equivalent pattern was observed during the second phase of prednisolone titration with a rapid decline in CRP during high prednisolone dosage and an increase shortly after tapering the dosage, with observation of a high CRP value at the relapse of clinical signs (day 143–160) (Fig. 1). The CRP declined to immeasurable values during the period of severe bone marrow suppression, where inflammatory activity was impaired by a depletion of inflammatory cells.

Thus, a marked increase in CRP were observed on two occasions, related to the reappearence of clinical signs of polyarthritis.

## Discussion

Infections of *Ehrlichia spp*. (*Anaplasma spp*.) were reported as a cause of type II IMPA in dogs [13] where it is assumed that the infectious process provides an antigenic source for immune complex formation, and either the antigen or circulating complexes are deposited within the synovium to initiate inflammation by a type III hypersensitivity reaction [14]. The involvement of *A. phagocytophilum *in the present diagnosis was based on clinical signs, cytological findings and a positive antibody titer against *Ehrlichia equi *(*A. phagocytophilum *[11]), but a negative PCR for *Ehrlichia spp*. antigen was also obtained. However, negative PCR was reported to be common even after short antibiotic treatment [15].

In general, the therapeutic regimen of type II IMPA is directed against the infectious agent, where recovery can be seen upon clearance of the infectious agent [1,13]. However, supportive immunosuppressive therapy is often part of the regimen to help resolve the joint inflammation, using either prednisolone alone or in combination with cytotoxic drugs [1]. When clinical signs of polyarthritis have resolved, the dosages are gradually reduced and if signs return the dosage is increased [1].

The observations made in the present case suggest that serial measurements of CRP during immunosuppressive therapy of canine type II IMPA could be useful as an objective marker of inflammatory activity. This could have a positive effect on case management of type II IMPA by possibly enabling early assessment of treatment efficiency and detection of undesirable inflammatory activity, unbiased by treatment. C-reactive protein is non-specific, regarding the cause of inflammation. Thus, a decrease in concentration during treatment indicates a general down-regulation of inflammatory activity[3-5,16]. Whereas, an increase in CRP concentration during treatment indicates increased inflammatory activity [3,4,17], due to increased disease activity or presence of other secondary stimulus (both information of clinical importance). Finally, canine CRP was reported not to be biased as a marker of inflammatory activity by neither steroid nor non-steroid drugs [6,7,18]. The WBC and neutrophil count in the present case were, however, adversely biased as inflammatory markers by the steroid treatment, as would be expected [2], and thus, the observed fluctuations of WBC and neutrophil count were not reflecting disease activity in a clinical useful manner. Canine serum CRP has earlier been studied as a marker of disease-activity in osteoarthritis (OA) [19], where only slightly elevated levels were observed. However, a marked difference between the CRP levels in a localized process (as OA [21]) and a generalized inflammatory disease (as IMPA in the present case), is expected, as CRP is a marker of systemic inflammation (produced in the liver upon stimulation by an increased level of circulating pro-inflammatory cytokines[20]).

In conclusion, CRP measurements seemed potentially valuable as an unbiased marker of the inflammatory activity during immunosuppressive treatment of type II IMPA in the present case, seemingly providing clinically useful information superior to that of CBC and clinical observations. Further studies should be conducted to investigate the general applicability of CRP as an unbiased marker of inflammatory activity during treatment of IMPA.

## Competing interests

The author(s) declare that they have no competing interests.

## Authors' contributions

GAH, LRJ and ATK were responsible for patient-management throughout the period of diagnosis and treatment. MKH and ALJ were responsible for the clinical pathological analyses and study design regarding CRP. All authors have been significantly involved in interpretation of data and drafting the manuscript. All authors have given final approval of the manuscript.
